# Prospects for Future Methodological Development and Application of Magnetoencephalography Devices in Psychiatry

**DOI:** 10.3389/fpsyt.2020.00863

**Published:** 2020-08-21

**Authors:** Naruhito Hironaga, Yuichi Takei, Takako Mitsudo, Takahiro Kimura, Yoji Hirano

**Affiliations:** ^1^Brain Center, Faculty of Medicine, Kyushu University, Fukuoka, Japan; ^2^Department of Psychiatry and Neuroscience, Gunma University Graduate School of Medicine, Maebashi, Japan; ^3^Department of Neuropsychiatry, Graduate School of Medical Sciences, Kyushu University, Fukuoka, Japan; ^4^Institute of Liberal Arts and Science, Kanazawa University, Kanazawa, Japan

**Keywords:** magnetencephalography, psychiatry, resting state networks, cortico-subcortical networks, optically-pumped magnetometers

## Abstract

Magnetoencephalography (MEG) is a functional neuroimaging tool that can record activity from the entire cortex on the order of milliseconds. MEG has been used to investigate numerous psychiatric disorders, such as schizophrenia, bipolar disorder, major depression, dementia, and autism spectrum disorder. Although several review papers on the subject have been published, perspectives and opinions regarding the use of MEG in psychiatric research have primarily been discussed from a psychiatric research point of view. Owing to a newly developed MEG sensor, the use of MEG devices will soon enter a critical period, and now is a good time to discuss the future of MEG use in psychiatric research. In this paper, we will discuss MEG devices from a methodological point of view. We will first introduce the utilization of MEG in psychiatric research and the development of its technology. Then, we will describe the principle theory of MEG and common algorithms, which are useful for applying MEG tools to psychiatric research. Next, we will consider three topics—child psychiatry, resting-state networks, and cortico-subcortical networks—and address the future use of MEG in psychiatry from a broader perspective. Finally, we will introduce the newly developed device, the optically-pumped magnetometer, and discuss its future use in MEG systems in psychiatric research from a methodological point of view. We believe that state-of-the-art electrophysiological tools, such as this new MEG system, will further contribute to our understanding of the core pathology in various psychiatric disorders and translational research.

## Introduction

Numerous research studies in the field of psychiatry have made use of magnetoencephalography (MEG) systems. Schizophrenia (SZ), major depression, autism spectrum disorders (ASD), child psychiatry, attention deficit hyperactivity disorder (ADHD), obsessive-compulsive disorder (OCD), dementia, and other conditions have been investigated using MEG either alone or in combination with multiple neuroimaging modalities. Such studies have commonly used auditory stimulation owing to the strong relationship between auditory system abnormalities and psychiatric disorders [e.g., auditory hallucinations in SZ (1)]. In particular, the auditory steady-state response (ASSR) and mismatch negativity (MMN) response are representative markers used to indicate auditory processing abnormalities in neurophysiological studies in psychiatry (2–7). Recently, research into resting-state networks (RSNs) has received much attention in both neuroscience and psychiatric research because RSNs play a key role in the baseline or default mode of normal and disordered brains. In psychiatric studies, evaluation of whole-brain dynamics would be of more interest than a functional localization-based approach (8, 9). Thus, time-frequency analysis, which is used to evaluate whole-brain activity, is frequently used in this field. The main advantage of MEG is its high temporal resolution and adequate spatial resolution.

**Figure 1:**
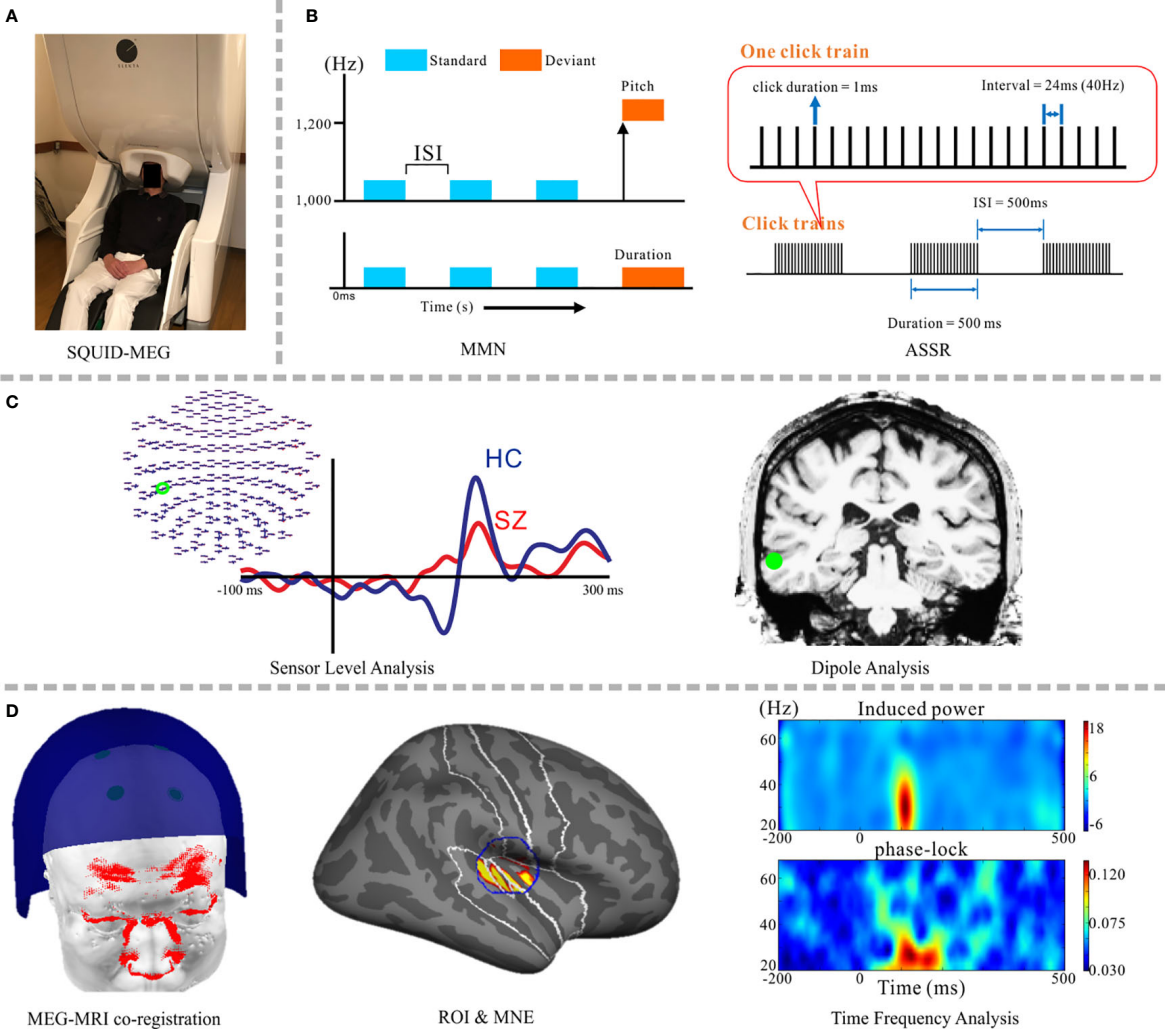
Schematic images from psychiatric research implementing the SQUID-MEG system. **(A)** SQUID-MEG system: the brain is covered by fixed positional sensors in the dewar helmet. **(B)** A representative, frequently-employed auditory stimulation paradigm for MEG diagnosis, including the MMN (left) and ASSR (right) responses. **(C)** Orthodox MEG analysis: a sensor-level analysis making a comparison between patients with SZ (red) and HC (blue) (left) and dipole analysis (right). **(D)** The current method used in MEG psychiatric research, from right side: accurate MEG-MRI co-registration using a 3D laser scanner (13), specified ROI and source reconstruction results using MNE, and time-frequency analysis using source waveforms; induced power (upper) and the phase-locking factor (lower). SQUID, superconducting quantum interference device; SZ, schizophrenia; HC, healthy control; MMN, mismatch negativity; ASSR, auditory steady-state response; ROI, region of interest; MNE, minimum norm estimates.

**Figure 2:**
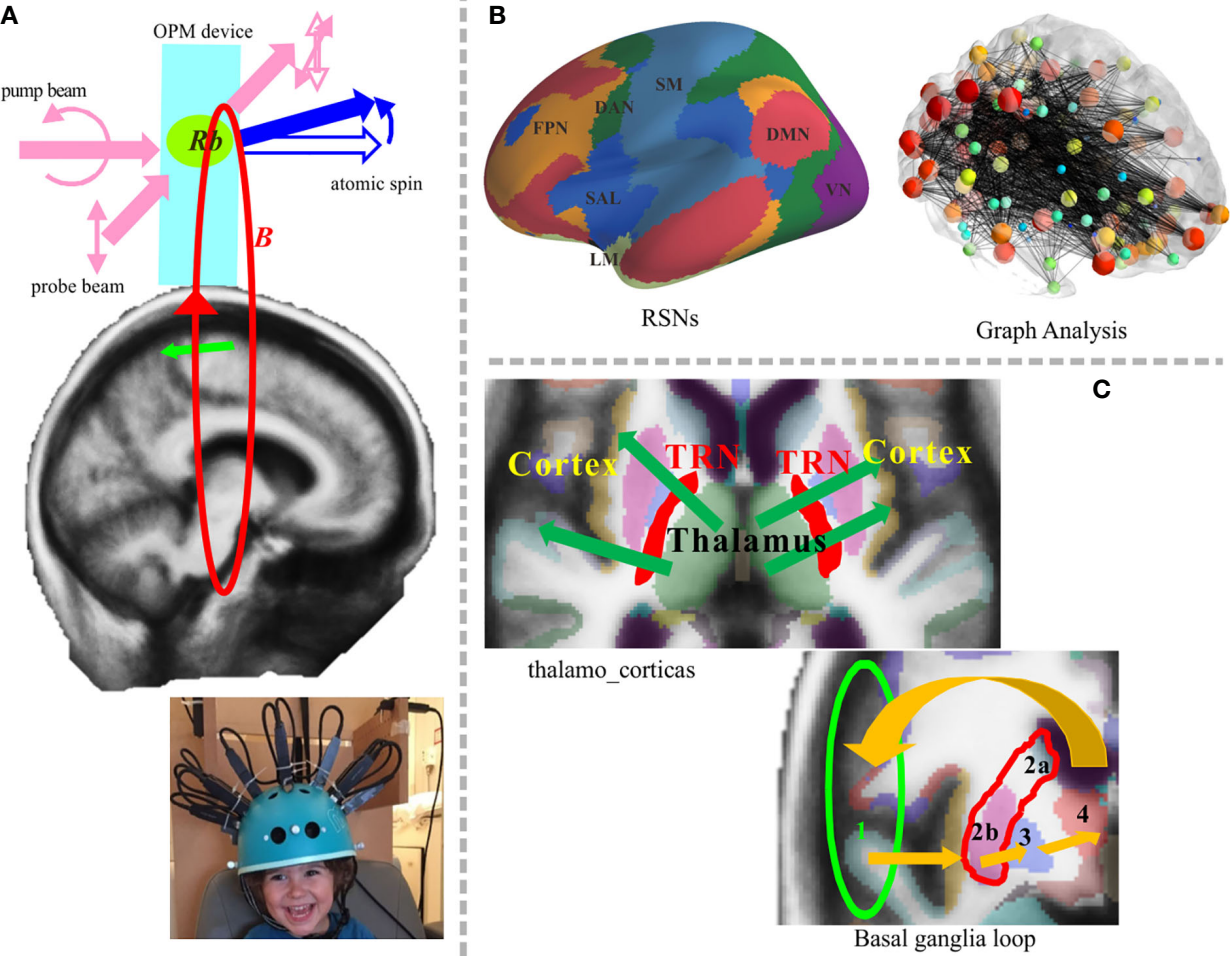
Potential application of the OPM-MEG system in future work. **(A)** Newly developed system and principle of atom magnetometer (upper panel). Two laser beams are arranged orthogonally; one is the pump beam that polarizes the vaporized atomic electron spin, and the other is the probe beam that measures the state of spin evolving in the magnetic field *B*. Rb denotes rubidium atom. The lower sub-figure presents a pediatric OPM-MEG system adapted from Hill et al. (14). **(B)** RSN analysis will involve deep brain structures. Representative RSNs (left) and an image showing graph theory analysis (right) (15). **(C)** The cortico-subcortical network could be targeted in future work. Thalamocortical communication (upper left) and the basal ganglia loop, 1. cortex→2. striatum (a. caudate nucleus orb. putamen)→3. pallidum→4. thalamus→1.cortex (lower right). OPM, optically pumped magnetometers; TRN, thalamic reticular nucleus.

Historically, detecting electromagnetic brain activity has been a topic of interest since the mid-20th century. Hans Berger, who invented electroencephalography (EEG), was a pioneer in methods of recording electrical brain activity. Subsequently, 30 years later, Baule and McFee successfully detected the heartbeat *via* magnetic field detection (10). Then, the superconducting quantum interference device (SQUID)-type magnetometer integrated with shield technology successfully achieved the detection of very small magnetic fields from the human brain (11). In the 1970s, the gradient-type magnetometer was developed (12) and multi-channel recording came into use in clinical settings in the 1980s. In the 1990s, whole-head-type MEG was developed by several vendors. At the beginning of the 21st century, no notable hardware development was taking place; however, the last decade has seen the development of new technologies. Thus, it is now pertinent to consider how new MEG technologies will contribute to psychiatry research. We first briefly summarize the basic principle of MEG and its use in psychiatric research. Next, we will describe the use of MEG in child psychiatry, the study of RSNs, and the study of cortico-subcortical networks. We also present schematic images comparing the current (**Figure 1**) and new MEG systems (**Figure 2**). Then, we will discuss the potential for these new MEG technologies to be used in future psychiatric research.

## MEG and Other Neuroimaging Modalities

In humans, several functional brain measurement techniques are now available. For example, functional magnetic resonance imaging (fMRI), near-infrared spectroscopy (NIRS), EEG, and MEG. Here, we will briefly describe the pros and cons of each technique when applied to psychiatric research. An advantage of MEG is notably its non-invasiveness, which is critical for psychiatric patients, while the strong magnetic field generated during fMRI recordings is questionably invasive especially for children and infants. Using blood oxygenation level-dependent signal changes, fMRI provides high spatial resolution if participants remain still but with a lower temporal resolution that limits its functional estimations. Moreover, artificial sound noise generated by the MRI device causes problem for auditory experiments, which are a major focus in psychiatric research. EEG exhibits high temporal resolution, equivalent to MEG, yet its spatial resolution is disputed due to distorted detection through several tissues with different conductivities. In psychiatric experiments, rapid and comfortable recordings that are related to preliminary processes and limitation of motions are ideal for patients. Preparation of EEG (i.e., fixing many electrodes) is time consuming. Alternatively, NIRS does not require a complex preparation process and is relatively robust to the problem of body movements, but it has less spatial and time resolution compared with EEG/MEG. Although the spatial resolution of MEG is disputed because of its ill-posed inverse solution, MEG can provide high temporal resolution under certain spatial resolution, enabling almost the whole cortex to be covered by any measurement in a single system. Indeed, being non-invasive, fast, and comfortable, MEG measurements offer several advantages for brain response recordings from psychiatric patients.

## MEG Analysis and Fundamental Frameworks

### MEG Analysis for Psychiatric Research

MEG sensors detect magnetic field changes in neuronal electric currents that are sensitive to the currents perpendicular to the sulci or fissures over the cortex. MEG source reconstruction accuracy depends on factors such as the forward and inverse problems, the signal-to-noise ratio (S/N) including the number of trials for averaging, and MEG-magnetic resonance imaging (MRI) co-registration issues. The first factor, the inverse problem, has been extensively addressed; however, other factors are crucial in practice. The patented methods of noise cancellation are applied as necessary, which include gradient formation (16) and the offline Maxfilter (17), which can help to achieve a higher S/N ratio. As a preliminary step, a band pass filter is applied to the frequency range of interest. Furthermore, independent component analysis (ICA) is a powerful tool for extracting target components or eliminating artifacts (18). We must then consider the MEG source reconstruction process. Given the widespread use of MRI, the co-registration process is now crucial for MEG, EEG, and even for transcranial magnetic stimulation (19). Stylus magnetic-field digitizers are commonly used. However, these are less accurate and more time-consuming than photogrammetry-based systems, 3D laser/structured-light scanners, or 3D printers, which are reliable alternatives for measuring head shape (13, 20–22). Once MEG-MRI alignment is fixed, forward computation needs to be considered. Currently, a realistic model utilizing the boundary element method (23) or the finite element method (24) is a popular and accurate approach. Following the lead field computation, the inverse solution should be applied to obtain a source activation map. Typically, we distinguish two groups of algorithms; one is the equivalent current dipole (ECD) (25), and the other is distributed source analysis. Currently, the minimum norm-based approach or an adaptive beam-former is common algorithms for the latter case (26, 27). Minimum norm estimates (MNE) is a technique that uses pre-fixed source points over the brain to minimize the total current of all nodes. Furthermore, a noise normalization technique is frequently applied using the pre-trigger period or entire raw data (28). Then, time frequency analysis should be applied using signals extracted from target regions. In psychiatric research, oscillatory analysis enables us to detect abnormal fast and varied neural activities, hence, time-frequency analysis and connective analysis including coherence, phase synchrony, and envelope correlation *via* wavelet transform, are commonly performed (29, 30). Recently, cross-frequency coupling, such as phase-amplitude coupling between low and high frequency oscillatory components, has also been discussed (31–34).

### Fundamental Frameworks

**Figure 1** depicts a schematic image of psychiatric research using SQUID-MEG. Sensors are fixed in the dewar (**Figure 1A**). Components elicited in response to auditory stimuli, the MMN and ASSR, are frequently used to detect auditory sensory deficits in psychiatric disorders (**Figure 1B**). Combination studies with sensor-level and dipole analysis have been popular (**Figure 1C**) (35–38). Hirano and colleagues demonstrated oscillatory deficits in response to speech sounds in SZ (39) and bipolar disorder (40), ASSR gamma oscillatory abnormalities in SZ (41) and mood disorders (42), and MMN deficits in major depressive disorder (43). Given the relatively high spatial resolution, distributed source analysis has become the main method for analyzing MEG data (**Figure 1D**) [e.g., MNE (44–46), the beamforming (47)]. At present, one state-of-the-art technique is applying wavelet transform and connected analysis to the extracted source waveforms *via* a combination of distributed source analysis and ICA (48, 49).

## Specific MEG Applications for Psychiatric Disorders

Here we describe three specific MEG applications in psychiatric disorders since we consider that these will be the key roles of the coming psychiatric study using MEG.

### Child Psychiatry

Owing to its completely non-invasive approach, MEG is one of the most suitable technologies in child psychiatry. Indeed, ASD and ADHD in child psychiatry have been well investigated using MEG (50–54). Although infant MEG systems are commercially available (55), only a few institutions possess these systems. The main obstacle to MEG use in babies and children is head and body movements. The simplest way to deal with this issue is to discard the data from when patients make large movements. Another way to overcome these issues is the use of continuous head localization; however, the use of strong magnetic field generation during the measurement in children is controversial. MRI scanning in younger children is problematic, but the use of standard brains from a series of age-matched infant MRIs can help to reduce the problem. Dipole methods with a spherical model are still the dominant approach in children; hence, the accuracy level in children is not comparable to that in adult cases.

### Resting-State Networks

Brain activity occurs not only during cognitive processes, but also during sleep or rest (56, 57), which is when RSN activity can be recorded. RSNs involve key network domains that are known to underlie the pathophysiology of many psychiatric disorders (58, 59), such as the default mode network, central executive network, and saliency network. Several RSN studies utilizing MEG for psychiatric researches have been reported (60–67). A common way of making use of the strengths of MEG is to investigate RSNs on the basis of a source reconstruction technique in various frequency bands. Analyzing frequency-specific RSNs involving deeper brain such as limbic regions can reveal pathophysiology of psychiatric disorders as network failure.

### Thalamocortical Communication and the Basal Ganglia Loop in Psychiatric Disorders

Recently, aberrant oscillatory activity has been reported in various psychiatric disorders. Functional interactions within the cortico-subcortical network are considered to contribute to abnormal oscillation *via* alterations in neurotransmitters and receptors in psychiatric disorders. We argue that there are relationships among the cortico-subcortical network (including the limbic system, thalamus, and basal ganglia) and related networks, which form functional loops that are of interest in psychiatric research. Although the ability of MEG to detect the activity of deep brain structures is still disputed, there are a few credible reports under current SQUID-MEG system (68, 69). Schulman et al. hypothesized that the generation of abnormal recurrent neuronal activation in neuropsychiatric disorders occurred through aberrant thalamocortical rhythm epiphenomena (70). Patients with OCD usually recognize that obsessions and compulsions are not logical, but they are not under their control; thus, it is important to investigate whether cognitive changes in OCD may be caused by abnormal neural circuits. The basal ganglia loops not only underlie sensorimotor control but also cognitive functions associated with limbic control and motivated behavior (71); hence, recent work on OCD has been focused on the contribution of the basal ganglia loops. fMRI studies have suggested that OCD involves abnormal functioning in specific frontal-subcortical brain circuits (72). Enhanced activity within the basal ganglia loop is clearly seen in OCD even at resting state (73). However, the temporal dynamic of these loops has not been sufficiently addressed in humans. The new MEG could help to elucidate the contribution of altered basal ganglia loops to OCD.

## Limitations of the Current SQUID-MEG System

MEG is a very attractive tool for psychiatric research, including in the diagnosis of psychoses. However, the penetration rate of MEG is generally very low compared with fMRI, EEG, and transcranial magnetic stimulation (58). In general, an MEG laboratory is expensive to set up and maintain. Further, despite MEG being simple to measure, it requires a complex data analysis. The source reconstruction analysis *via* a mathematically ill-posed problem requires a complex knowledge of physics, mathematics, and computational issues. Current practical limitations of SQUID-MEG include its poor sensitivity to deep brain activities and its sensitivity to head motion artifacts. These problems are not solvable in principle because they depend on the hardware design and configuration. The main hardware limitations of the SQUID-MEG system relate to the fixed location and orientation of the detection coils (sensors). The fixed coil position within the dewar limits the distance between the brain and the sensors to approximately 2−3 cm and the gradiometers (axial or planar) are designed to increase the sensitivity of the cortical responses. Indeed, using the current SQUID-MEG system, it is difficult to detect activity in the thalamus and basal ganglia accurately because of their depth and anatomical structure. Additionally, the fixed coil location requires the subject to lie still during measurement. As such, the system is sensitive to head movement. These limitations of the current SQUID-MEG system reduce its utility in child psychiatry and analysis of cortico-subcortical networks. Thus, there is increasing interest in the development of new MEG systems, as described below.

## Newly Developed MEG Sensors

Until the 20st century, low temperature SQUID-MEGs were the only choice of device for MEG sensors; however, current technologies have meant that new MEG devices are now available. In particular, the development of optically-pumped magnetometers (OPM; also termed ‘on-scalp MEG’) has accelerated in recent years. The development of the atomic magnetometer began earlier, but because of their poor sensitivity, their use was not emphasized. Drastic changes have occurred since the development of a new spin-exchange relaxation-free condition, which has led to sensitivities up to the femtotesla range (74). **Figure 2** shows potential uses of OPM-MEG in future research. **Figure 2A** (upper) depicts a schematic image of the fundamental principle of an atomic magnetometer embedded in an OPM device. Studies using this new technology have reported results that are relevant to psychiatric research, such as phase-locked evoked responses generated by auditory stimuli (75) and the detection of induced changes in the frequency domain (76). One of the most important issues when considering the newly developed OPM is the wearable-type system, which increases the flexibility of sensor location and orientation. The on-scalp MEG system can reduce the distance between the sensors and the brain, allowing detectors to be placed several millimeters above the scalp surface. The flexible position and orientation indicate that this system could be used to measure activity of not only the cerebral cortex but also subcortical brain areas. The S/N ratio will be drastically improved which is not comparable to SQUID-MEG. A study from 2017 reported that the S/N ratio was four times higher than the SQUID-type MEG in general, and eight times higher on surface areas (77). By using flexible-type sensors, it is possible to freely orientate the sensors according to the target brain structure. When using wearable-type sensors, MEG-MRI co-registration is crucial because of non-fixed flexible sensor locations and orientations, but some proposals have been made for the use of OPM with 3D printers or 3D-structured light scanners (21, 22, 77). The higher S/N ratio and the orientation-free design of the system allows the detection of deeper brain signals. The accuracy of source localization-based time-frequency analysis also ameliorated along with the hardware development. Another advantage of wearable-type sensors relates to the reduced influence of head movements during measurement. Notably, the maintenance cost of MEG will be drastically reduced because the new sensor works at room temperature. We strongly believe that this non-pyrogenic MEG device will increase the penetration rate of MEG.

## Utilization of OPM-MEG for Psychiatric Research

The OPM-MEG could be a better, more accurate alternative method for recording neuromagnetic signals from infants and children since the wearable sensors reduce the problem of head movement compensation (**Figure 2A**, lower). A higher S/N ratio would also allow single-trial analyses to be performed, which is beneficial to detecting the dynamic changes in the brain. For example, MMN experiments generally consist of standard and deviant stimuli, and the number of deviant stimuli is lower than the number of standard stimuli. As such, there is an incongruence in the number of averaged trials between the two conditions. Thus, the interest around single-trial analysis of OPM-MEG data in MMN research is increasing. Oscillatory whole-brain analyses in milliseconds resolution is a good tool for RSN analysis (**Figure 2B**). Covering the whole cortex is crucial in psychiatric research because the RSNs and recurrent loops are likely to be important in psychiatric diseases, as described in the previous subsections. The possibility of using OPM to detect the activity of deep brain structures has not yet been examined systematically. Nevertheless, OPM was suggested to detect deeper source signals because of its higher S/N ratio and orientation-free system. Indeed, a recent OPM-MEG study reported that the detection accuracy for deeper source signals is almost twofold that of SQUID-MEG and even allowed for detection in the spinal cord (77). However, further advances are required for assessment of cortico-subcortical networks. In recent years, there have been systematic advances in the SQUID-MEG system. With respect to hardware, developments in gradiometers, magnetometers, and reference channels have improved the detection of both shallow and deep sources, which may be useful for cortico-subcortical network analysis. From a theoretical viewpoint, an optimized weighted lead field matrix for deeper source detection, and integration of signal separation methods such as ICA, would also aid in cortico-subcortical network analysis. However, the practical development of such sophisticated subcortical-level network analyses for the new MEG system will take a little more time. Thus, it may be better to initially focus on the shallow source activity because these subcortical networks involve cortical signals. Then, we can shift to the measurement of deeper brain signals and development of more sophisticated tools and devices. Finally, advanced task configuration and experimental design should be considered for reliable measurement of activity in deeper brain networks. Although OPM-MEG can detect activity in deeper brain regions, its credit level is not identical to fMRI. Nonetheless, OPM-MEG provides valuable temporal information. At any rate, the possibility of deep-brain recordings would represent a considerable advancement compared with the current scenario. Detection of deeper brain structures using OPM-MEG enables measurement of thalamocortical communication and the basal ganglia loop, which are of great interest in psychiatric research (**Figure 2C**). Given the importance of temporal information and the limitations of fMRI, RSN analyses will be extended to the wider brain area, with the possibility of exciting temporal information.

## Conclusion

We addressed the limitations of the current SQUID-MEG system, the expectations of the newly developed MEG system, and how this system should be utilized in psychiatric research. Using MEG, investigation of brain dynamics with high temporal resolution will be invaluable for psychiatric research. Ideally, future investigations will incorporate data on the order of milliseconds that can be used to better understand the role of complex brain networks in psychiatry, such as those involving the cerebrum, cerebellum, limbic system, thalamus, basal ganglia, and their related networks. The development of sensors should provide new insights in child psychiatry and entire brain networks involving deeper structures. Although multimodal analyses are more powerful in principle, the newly developed OPM-MEG neurophysiological device will be very useful and accurate for psychiatric research, even as a standalone single system, because OPM-MEG has both higher temporal and spatial resolution. Note that development of such state-of-the-art functional neuroimaging systems will provide further advances in the fields of neuroscience and psychiatric research. Identifying novel biomarkers for detection of psychiatric disorders is a key goal of psychiatric research (2, 3, 9). We believe that continued MEG advancement will open new doors for establishing neurophysiological biomarkers for psychiatric disorders in the near future.

## Data Availability Statement

All datasets presented in this study are included in the article/supplementary material.

## Ethics Statement

The studies involving human participants were reviewed and approved by Ethics Committee of the Graduate School of Medical Sciences, Kyushu University. The patients/participants provided their written informed consent to participate in this study. Written informed consent was obtained from the individual(s) for the publication of any potentially identifiable images or data included in this article.

## Author Contributions

All authors, NH, YT, TM, TK, and YH, have contributed to writing the manuscript.

## Funding

This work was supported by Scientific Research from the Ministry of Education, Culture, Sports, Science, and Technology; Grant-in-Aid for Young Scientists (B) (no. JP16K19748) and for Scientific Research (A) (no. JP19H00630), (B) (nos. JP17H02624 and JP19H03579) and (C) (nos. JP19K08038, JP18K07604, and JP20K12572); AMED under grant (no. JP20dm0207069); and SIRS Research Fund Award (YH) from Schizophrenia International Research Society.

## Conflict of Interest

The authors declare that the research was conducted in the absence of any commercial or financial relationships that could be construed as a potential conflict of interest.
